# Rerupture after flexor tendon repair of the hand and wrist: a retrospective risk factor analysis

**DOI:** 10.1007/s00402-026-06302-7

**Published:** 2026-05-02

**Authors:** Sarah Franziska Marwieser, Stella Vavricka, Peter Kaiser, Gernot Schmidle, Richard Andreas Lindtner, Rohit Arora, Marko Konschake

**Affiliations:** 1 Medical University of Innsbruck, Innsbruck, Austria; 2 Institute for Clinical and Functional Anatomy, Medical University of Innsbruck, Innsbruck, Austria; 3 Department of Orthopedics and Traumatology, Medical University of Innsbruck, Innsbruck, Austria; 4 Sportclinic Arlberg, St. Anton, Austria; 5 Schulthes Clinic, Zürich, Switzerland

**Keywords:** Flexor tendon, Hand surgery, Reruptures, Secondary ruptures, Suture ruptures, Predictors

## Abstract

**Introduction:**

Rerupture after primary flexor tendon repair of the hand and wrist is a serious complication that can substantially impair hand function and restrict activities of daily living, occupational performance, and recreation. Identifying factors associated with rerupture is essential to optimize surgical techniques and postoperative rehabilitation strategies and to improve patient outcomes.

**Methods:**

A retrospective observational cohort study was conducted including patients who underwent surgical repair of flexor tendon injuries of the hand and wrist at a single institution between 2010 and 2022. The study included 292 patients with 429 complete tendon injuries. Collected data comprised demographic characteristics, injury mechanism and type, anatomical location, surgical timing and technique, and postoperative immobilization and rehabilitation protocols. Associations between potential risk factors and rerupture were explored using univariable analyses, followed by a multivariable logistic regression model to identify independent predictors.

**Results:**

The overall rerupture rate was low but clinically relevant, occurring in a small proportion of patients and tendon lesions. Rerupture was more frequently observed in male patients, older individuals, and those with specific injury mechanisms. Surgical factors, particularly the need for pulley reconstruction, were associated with a higher risk of rerupture. All reruptures occurred in patients who underwent prolonged immobilization. In the multivariable analysis, male sex and pulley reconstruction remained independent predictors of rerupture, whereas rehabilitation-related variables did not independently influence risk.

**Conclusion:**

This large single-center cohort study identifies male sex and pulley reconstruction as independent risk factors for rerupture following primary flexor tendon repair. Awareness of these factors may assist surgeons and therapists in patient counseling, risk stratification, and the development of tailored postoperative strategies. Prospective multicenter studies are warranted to confirm these findings and further refine preventive approaches.

**Level of evidence:**

Level III.

## Introduction

Flexor tendon lacerations of the hand and wrist frequently present in the emergency department and usually require surgical repair. Complications such as tendon rerupture after primary suture repair and adhesion formation due to postoperative scarring remain major challenges. These issues often lead to prolonged rehabilitation, reduced functional outcomes, and a significant socioeconomical burden.

Tendon rerupture typically necessitates secondary surgical intervention. Depending on the time of reruptre, re-suturing may be immediately feasible. However, outcomes after revision repair are significantly worse compared to primary repair [1–3]. Preventing rerupture is therefore of paramount importance for optimizing functional recovery and preserving quality of life. Identifying the underlying causes of rerupture and determining modifiable risk factors are essential steps toward improving clinical outcomes.

Previous studies have investigated a range of potential risk factors, including patient demographics, injury characteristics, surgical techniques, and postoperative rehabilitation protocols. Some reports suggest that male sex, advanced age, and delayed surgical intervention may increase the risk of rerupture. However, most of these findings are based on small cohorts, which limits statistical power and provides tendencies rather than robustly significant results [1, 2, 4–16].

The present study aims to address these limitations by analyzing a large retrospective patient cohort to identify risk factors of flexor tendon repair failure. By systematically evaluating patient-related, injury-related, surgical and postoperative factors, this study seeks to generate clinically relevant insights that may help optimize surgical strategies and rehabilitation protocols, ultimately improving patient outcomes.

## Materials and methods

### Study design

This retrospective, single-center cohort study was conducted at a European university hospital. Patients treated for flexor tendon lesion of the hand and wrist between January 1, 2010, and October 16, 2022, were included. Ethical approval was obtained from the local ethics committee (Nr. 1404/2022).

### Patient cohort

The study cohort comprised 465 patients with tendon injuries, consisting of 137 patients (276 tendons) with partial tendon lesions and 292 patients (429 tendons) with complete tendon lesions (Table 1).

Partial lesions were evaluated descriptively only, as rerupture occurred in only 1 of 276 tendons (0.4%), making risk factor modeling inappropriate. Consequently, inferential risk factor analysis was confined to patients with complete tendon lesions. These complete lesions affected one or more flexor tendons and were surgically repaired using core and/or peripheral suture techniques. The subgroup of patients with complete lesions included 216 males and 76 females, with a mean age of 37.6 ± 18.6 years (range, 1–93 years).

**Inclusion criteria** comprised patients with primary flexor tendon repair in all anatomical zones and involving the following tendons: flexor pollicis longus (FPL), flexor digitorum profundus (FDP), flexor digitorum superficialis (FDS), flexor carpi radialis (FCR), and flexor carpi ulnaris (FCU).

**Exclusion criteria** included amputations and incomplete medical records.

### Variables and data collection

Patient data were retrieved from a prospectively managed electronic health record system (Cerner^®^ PowerChart^®^, Cerner Millenium©, Cerner Corporation 2011).

The following variables were extracted:

- **Demographics**: age (≤ 18, 19–29, 30–49, 50–69, ≥ 70 years), sex (female, male).
- **Injury characteristics**: date of injury, date of first examination, date of surgery, mechanism of injury (cut/stab, crush, saw, axe, hyperextension, chronic); open vs. closed injury; side (right/left), finger (thumb, index, middle, ring, little finger, wrist),

tendon (FDP, FDS, FPL, FCR, FCU), and anatomical zone affected (1–5); number of tendons and rays involved (single/multiple); complete vs. partial lesions (with percentage in partial lesions); associated injuries (Yes/No).

- **Surgical procedure**: timing (classified as acute primary repair, day 0–1; delayed primary repair, day 1–14; secondary repair, weeks 3–5; and late secondary repair > 5 weeks), duration (< 30 min, 30–120 min, > 120 min), anesthesia type (General, Regional, Wide-Awake Local No Tourniquet).
- **Suture techniques**: Core suture technique (2-strand [Kirchmayr-Kessler, Tsuge], 4-strand [U Tang], 6-strand [M-Tang], Other [Bunnell, Krackow, mattress stitch, Anchor, Transosseous], Not documented), Core suture material (Braided polyblend, Braided polyester, Other), Core suture diameter (≤ 3 − 0, 4 − 0, ≥ 5 − 0), Peripheral suture (Yes/No), Peripheral suture technique (simple running suture [SRS], simple locking suture [SLS], Other), Peripheral suture material (Nonabsorbable monofilament, Other), Peripheral suture diameter (≤ 5 − 0/≥ 6 − 0), Execution of pulley reconstruction (Yes/No).
- **Team composition**: qualification of the main surgeon (Consultant/Resident), presence of a certified hand surgeon (Yes/No), and hand surgeon as part of the team regardless of qualification level (Yes/No).
- **Postoperative management**: Type of rehabilitation (Immobilization/Motion), Rehabilitation regime (Immobilization, Duran-Houser, Kleinert, early active motion, Not specified), and duration of cast/splint (1–5 weeks, 6 weeks, 7–8 weeks).
- **Rerupture analysis**: Total patients (reruptured tendons) by interval (≤ 6 weeks, 7–12 weeks, > 12 weeks), Causes by interval after primary repair (Incompliance, Incompliance + complex injury + trauma, Incompliance + infection, Iatrogenic, Complex injury, Trauma, Minor trauma with rheumatic disease, Increased scarring). Incompliance was defined as documented deviation from the prescribed postoperative rehabilitation protocol, such as premature removal of the splint, excessive active motion, or missed therapy sessions.

### Statistical analysis

Associations between rerupture rates and individual variables were analyzed using contingency tables. Depending on table size and expected cell counts, Chi-square tests, Fisher’s Exact Test, the Likelihood Ratio test, or the Fisher–Freeman–Halton Exact Test were applied as appropriate.In a second step, variables associated with rerupture in univariable analysis with a significance level of *p* < 0.05 were included in a binary multiple logistic regression model to identify independent predictors for rerupture. The category with the lowest rerupture rate but with more than zero events was used as the reference group, a p-value < 0.05 was again considered significant.

## Results

During the study period, a total of 705 tendons were surgically repaired in 465 patients. Of these, 137 patients (276 tendons) presented with partial tendon tears, while 292 patients (429 tendons) sustained complete tendon lesions.

### Partial flexor tendon lesions

Partial tendon lesions were excluded from further analyses due to the extremely low rerupture- rate in this subgroup (1/276 tendons, 0.4%). Among these partial tears, the most common extent of lesion was 50% (25.7%), followed by 90% (10%). Notably, the single case of rerupture occurred in a tendon with a 90% lesion. Anatomically, partial tears were most frequently located in Zone 2 (41%) and Zone 5 (28%). Zone 1 was affected in 24% of cases, whereas Zones 3 and 4 were less frequently involved (7.3% and 0.4%).

The tendons most often affected in partial lesions were the flexor digitorum profundus (FDP, 45%) and flexor digitorum superficialis (FDS, 32%). No associated injuries were documented in 52.8% of cases. When present, the most common associated injuries were nerve injuries (18%), palmaris longus injury (8%), and vascular injuries (7.6%). All subsequent evaluations and statistical models focused exclusively on complete tendon lesions (Table 1).

### Complete flexor tendon lesions

The overall rerupture rate after repair of complete flexor tendon injuries of the hand and wrist was 21 of 429 tendons (4.9%). To identify potential risk factors for rerupture, we analyzed demographic factors, injury characteristics, surgical procedure, suture technique, team composition, and rehabilitation regimes.

Considering the postoperative interval until rerupture, more than half of all reruptures (12 tendons, 57%) occurred within the first 6 weeks after surgery, during which a cast or splint is typically still worn. Six reruptures (29%) occurred between 6 and 10 weeks postoperatively, corresponding to the first month after removal of immobilization.

The remaining three reruptures (14%) were observed between 6 and 12 months after surgery. Seven causes of secondary rupture were identified: non-compliance, acute complex injury of the affected area, trauma during the rehabilitation process, iatrogenic causes, infection, induration, and minimal trauma in the presence of an underlying rheumatic disease.

The most common cause—also contributing in the case involving two combined factors—was non-compliant behavior during the rehabilitation process, observed in 11 patients (52%). When seen in relation to the time of rupture (Table 2), non-compliance was found to play a role across all stages of rehabilitation.

### Patient-related factors

Male patients showed a higher rerupture rate than female patients (7.4% [16/216] vs. 1.3% [1/76]); however, this difference did not reach statistical significance (*p* = 0.082, Fisher’s Exact Test).

When analyzed by age, rerupture rates increased with advancing age across both sexes. The highest rerupture rate was observed in patients aged ≥ 70 years (12% [2/17]), followed by those aged 50–69 years (11% [7/65]). Overall, the association between age group and rerupture was statistically significant (*p* = 0.047, Fisher–Freeman–Halton Exact Test).

**Fig. 1:**
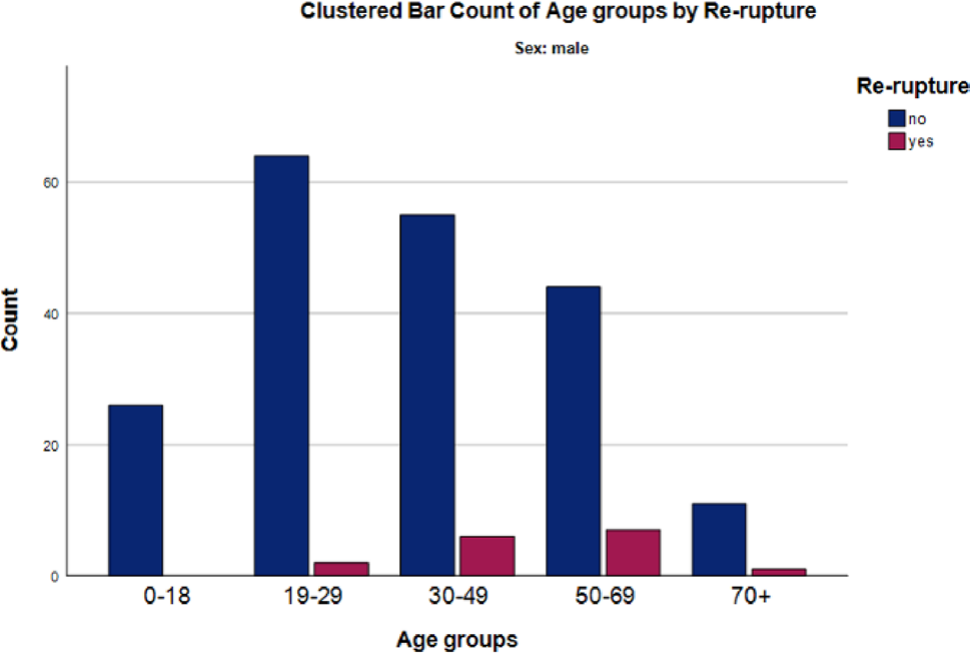
Rerupture rates after complete flexor tendon repair according to sex and age groups (male)

Among female patients, the only rerupture occurred in the ≥ 70-year age group (20% [1/5]); this association did not reach statistical significance (*p* = 0.066, Fisher–Freeman–Halton Exact Test). In male patients, the highest rerupture rate was observed in the 50–69-year age group (14% [7/51]), followed by the 30–49-year group (9.8% [6/61]), the ≥ 70-year group (8.3% [1/12]), and the 19–29-year group (3.0% [2/66]). The association between age group and rerupture in male patients did not reach statistical significance (*p* = 0.091, Fisher–Freeman–Halton Exact Test; Fig. 1).

### Injury-related factors

With the exception of injury etiology, none of the injury-related variables demonstrated a significant association with rerupture after complete flexor tendon repair (Table 3). Rerupture rates did not differ significantly with respect to hand side (left 5.1% [23/223] vs. right 4.6% [9/185], *p* = 0.823, Chi-Square Test), injury location (finger *p* = 0.775, Fisher-Freeman-Halton Exact Test; zone *p* = 0.614, Fisher-Freeman-Halton Exact Test; tendon type *p* = 0.596, Fisher-Freeman-Halton Exact Test), type of injury (closed 6.7% [2/30] vs. open 4.8% [19/399], *p* = 0.651, Fisher’s Exact Test), number of tendons and rays involved (single tendon 5.5% [11/200] vs. multiple tendons 4.4% [10/229], *p* = 0.657, Fisher’s Exact Test; single ray 4.0% [12/297] vs. multiple rays 6.8% [9/132], *p* = 0.231, Fisher’s Exact Test), or the presence of concomitant injuries (3.3% [7/215] vs. 6.5% [14/214], *p* = 0.115, Chi-Square Test).

Additional injuries were defined as concomitant nerve, vascular, bony, or muscular injuries, as well as injuries of the palmaris longus, extensor, adductor or abductor tendons, the pulleys, and joint dislocations.

In contrast, etiology showed a significant correlation with rerupture (*p* = 0.005, Fisher-Freeman-Halton Exact Test). While the vast majority of injuries were due to acute trauma (415, 97%), the highest rerupture rate was observed after axe injuries (9, 33%), despite being the rarest mechanism. Sharp object injuries represented the most frequent cause and carried a relatively low rerupture risk (4.3%, 13/305), whereas hyperextension (6.7%, 1/15) and crush injuries (9.7%, 3/31) were associated with higher rerupture rates. Injuries caused by saws (54, 13%), drills, or other rare mechanisms (10, 2.3%) resulted in no reruptures (0.0%).

Chronic injuries were defined as lesions caused either by underlying rheumatic diseases (2, 0.5%) or occurring after volar plate fixation (3, 0.7%) and were characterized by delayed diagnosis and/or prolonged symptom duration. Although rare (1.2%, 5/429), they were associated with a high rerupture rate of 20.0%, with the single rerupture in this group occurring in a patient with rheumatic disease.

### Surgery-related factors

**Fig. 2:**
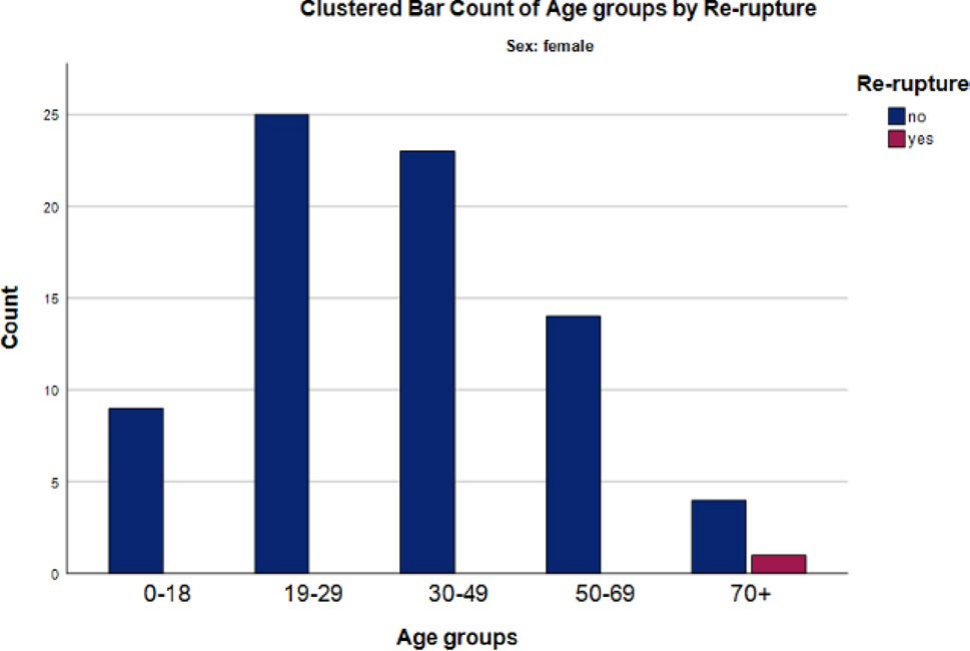
Rerupture rates after complete flexor tendon repair according to sex and age groups (female)

**Fig. 3:**
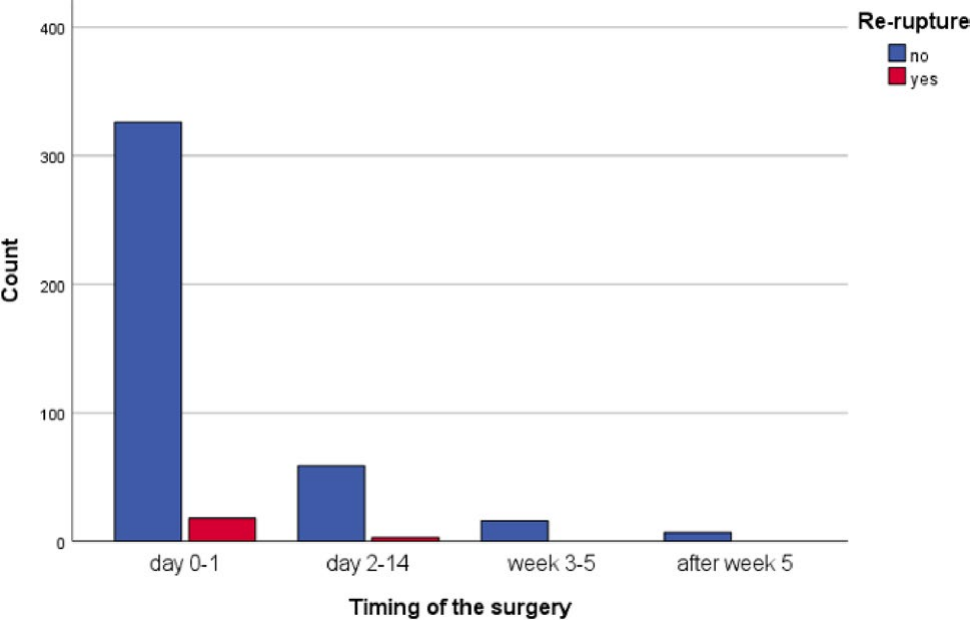
Bar chart of the time from injury to surgery

The median time from injury to surgery was 1 day (range 0-206, percentile 50 = 0), while the mean was 3 ± 13 days due to the influence of outliers. The majority of surgeries (80%, 344/429) were performed within one day after injury (Fig. 2). Patients who underwent acute primary repair had a higher, though not statistically significant rerupture rate of 5.2% (18/344) for surgery within one day, 4.8% (3/62) for surgery on days 2–14, and no reruptures (0/23) in patients operated in weeks 3–5 or after week 5; *p* = 1.000, Fisher-Freeman-Halton Exact Test (Table 4). In chronic tendon injuries, the mean time to surgery was 13.3 days (Fig. 3).

The median duration of the surgery time was 93 min (range 29–401, percentile 50 = 93), the mean was 122 ± 85 min. The case with the operation lasting less than half an hour had no occurrence of secondary rupture. The difference of occurrence of reruptures between surgery durations from half an hour up to two hours (4.3% [13/286]) in comparison to those of less than half an hour and to the ones lasting more than two hours (6.2% [8/121]) was not significant (*p* = 0.492, Fisher-Freeman-Halton Exact Test).

Rerupture rates were 6.1% (16/263) under general anesthesia and 5.8% (3/52) under regional anesthesia. The lowest rate was observed with wide-awake local anesthesia no tourniquet (WALANT) (1.8%, 2/114 tendons), suggesting a potential benefit of this technique; however, the differences were not statistically significant (*p* = 0.136, Likelihood Ratio, Table 4).

Rerupture rates did not differ significantly between procedures performed by residents (5.9% [6/95]) and consultants (4.6% [15/313], *p* = 0.599, Fisher’s Exact Test). Similarly, the presence of a hand surgeon on the surgical team, regardless of training level, had no significant effect on rerupture rates (4.7% [15/307] vs. 5.6% [6/101], *p* = 0.796, Fisher’s Exact Test) (Table 4).

The choice of core suture technique (*p* = 0.735, Fisher-Freeman-Halton Exact Test) and the use of a circumferential peripheral suture (*p* = 0.093, Fisher’s Exact Test) were not significantly associated with rerupture. In contrast, execution of pulley reconstruction was significantly associated with increased rerupture risk (*p* = 0.006, Fisher’s Exact Test, Table 5). Even though the distribution of the characteristics of core suture material differed significantly (*p* = 0.025, Fisher-Freeman-Halton Exact Test), in more than one third (37%, 157/429) of cases the documentation of the material used was missing. When solely looked at documented cases that either used braided polyblend or braided polyester, the rerupture rates did not differ significantly (*p* = 0.773, Fisher’s exact test) between these two groups. No definitive correlation with diameter (*p* = 0.816, Fisher-Freeman-Halton Exact Test) could be established due to missing documentation (Table 4). Peripheral sutures showed higher rerupture after simple running stitches (7.3%, 7/89) compared to simple locking stitches (1.6%, 1/63), though 177 cases (12 reruptures, 6.8%) lacked documentation and the difference was not statistically evaluable (*p* = 0.420, Fisher-Freeman-Halton Exact Test, Table 6).

### Rehabilitation factors

Regarding rehabilitation protocols, no statistically significant difference in rerupture rates was found between motion and immobilization regimes (*p* = 0.587, Fisher’s Exact Test) (Table 7). Patients managed with immobilization had comparable rerupture rates to those following passive motion rehabilitation (Duran-Houser and Kleinert) and early active motion (EAM) protocols (*p* = 0.673, Fisher-Freeman-Halton Exact Test). The choice of postoperative rehabilitation protocol was based on the suture technique used, patient compliance, and the individual assessment of the surgeon.

The median duration cast, or splint immobilization was 6 weeks (range 1–8, percentile 50 = 6), the mean was 6 ± 1 week. Only 17% (70/429) of lesions were treated with a cast or splint for less, 0.7% (3/429) for more than 6 weeks. No reruptures occurred within those two groups which was no significant difference to the rerupture rate of 5.9% (21/335) with the main group who wore the cast or splint for 6 weeks (*p* = 0.081, Fisher-Freeman-Halton Exact Test, Table 7).

### Logistic regression analysis

In the univariable analysis, age, injury etiology and the execution of pulley reconstruction were significantly associated with rerupture after complete flexor tendon repair of the hand and wrist. The association of these variables to the outcome was examined in a multivariable binary logistic regression model. The Nagelkerke R^2^ indicated that this model accounts for 26% of the variability in occurrence of rerupture. As for male patients the association of age and rerupture was as well significant, in a second step the variable sex was added blockwise to the regression model. The relationship between the predicators and the risk of rerupture resulted to be slightly stronger with a Nagelkerke R^2^ of 0.315 and the variable had a significant association (*p* = 0.035), therefore sex was retained in the model.

In the multivariable binary logistic regression model, male sex and pulley reconstruction emerged as independent predictors (OR = 13.235, *p* = 0.035; OR = 0.211, *p* = 0.004; Table 7). Age and injury mechanism did not remain statistically significant (*p* = 0.150; *p* = 0.375; Table 7).

## Discussion

In this study, we evaluated 29 potential risk factors for rerupture after primary flexor tendon repair of the hand and wrist. Among 292 patients with 429 complete lesions, the overall rerupture rate was 4.9% (males: 7.4%, females: 1.3%). This rate aligns with previously reported incidences, which range between 4% and 5% [3, 10–12, 17, 18].

Most reruptures occurred within the first 6 weeks, with patient non-compliance being the most frequently documented cause. In univariable analysis, sex, age, injury etiology and pulley reconstruction were significantly associated with rerupture. However, in multivariable analysis, only male sex and pulley reconstruction remained independent predictors of rerupture following flexor tendon repair. Although multivariable analysis is methodologically superior and identifies independent predictors, the relatively small number of events in our cohort may have limited statistical power. Thus, some true associations might not have reached significance in the multivariable model. Nevertheless, univariate results are therefore not meaningless; they provide valuable indications of potential risk factors and may serve as hypotheses for larger studies with higher event numbers, where they could become significant.

Our results demonstrate a rerupture rate of 7.4% in male patients, which is consistent with the findings of Harris et al., who reported a rate of 6% [11]. A large Swedish registry study including 1,372 patients identified male sex as an independent predictor of rerupture (*p* = 0.004), attributing this to a combination of epidemiological and behavioral factors [12]. In addition, a population-based study from England and Wales comprising more than 91,000 cases confirmed the predominance of male patients (75%) and reported rerupture and adhesions as the most frequent complication [19].This could be explained by the fact that men may experience greater tendon loading due to increased muscle mass and higher activity levels, which could place additional stress on the repaired tendon. Moreover, male patients may be more likely to return to strenuous activities earlier in the recovery period due to deliberate misconduct or unintentionally through lack of awareness, further increasing their risk of mechanical failure [2, 13]. The current literature suggests that patient compliance plays an important role in functional outcomes and the prevention of adhesions, but it is not regarded as the strongest or an independent risk factor for reruptures after flexor tendon repair. The main risk factors reported in previous studies are rather patient- and injury-related characteristics [12, 18, 20].

Nevertheless, patient compliance with the rehabilitation program primarily influences functional outcomes, whereas premature loading and general non compliance, whether due to intentional disregard or unintentional misunderstanding of postoperative instructions, may contribute to complications.

In the mulitvariate analysis it could also be confirmed that pulley reconstructions significantly increase the likelihood of reruptures.

This observation is consistent with the clinically observed increase in gliding resistance during intraoperative active testing, which in recent years has led to a shift toward targeted pulley release, including partial or complete release of the A2 and A4 pulleys when stable repair techniques permit early active rehabilitation [4–7]. Accordingly, optimizing tendon gliding should be prioritized, while pulley reconstruction may be reserved for secondary procedures in cases of clinically relevant bowstringing [21, 22].

Previous studies have identified age as an independent predictor for reoperation or rerupture after flexor tendon repair [11, 12]. Svingen et al. found that age over 25 years was a significant risk factor, Lalchandani et al. reported increased reoperation rates in patients aged 30–59, and Dy et al. observed that patients requiring reoperation were significantly older [12, 14, 16]. In contrast, in our cohort, reruptures in the univariate analysis occurred only in women over 70, while in men most cases were observed from age 30 onwards, and age was not statistically significant in the multivariate models. This may be due to the low number of cases in the respective subgroups, limiting statistical power.

Titan et al. describe age-related changes in tendon structure as a possible cause of impairments in tendon healing, which may in turn lead to reruptures. They note that changes in collagen fibril composition and alignment in the aging population can compromise the healing of flexor tendons [23].

The etiology of injuries has not yet been thoroughly investigated in the literature. In this study, acute initial injuries occurred most frequently as a result of cut or stab wounds. However, the highest rerupture rate was observed in patients whose injury was caused by an axe (33%). It could have been assumed that adhesions might impair the healing process in case of multiple tendons injured at the same ray or that the number of injured rays or the location of the injury (affected finger/wrist, anatomical zone, flexor tendon) influences the rerupture rate due to different motion mechanisms and healing processes [8, 23–30].

In the present study, the likelihood of rerupture is not statistically affected by any of these variables. This may be due to the consistent use of structured motion protocols in postoperative rehabilitation, which could have mitigated potential negative effects of more complex injuries, such as adhesions in cases of multiple tendon lesions. Alternatively, the lack of a statistically significant association might be explained by the relatively small sample size in relation to the high number of variables analyzed or influenced by specialized care provided in a FESSH accredited hand trauma center.

Hyperextension injuries are the most common closed flexor tendon injury, occurring when actively flexed DIP joints are forced into hyperextension, as often happens in ball sports [25, 31]. In this study, injuries typically occurred when fingers were caught on objects such as jackets or dog leashes. Chronic injuries were rare (*n* = 5) but showed a high rerupture rate of 20%, with the single rerupture occurring in a patient with rheumatic disease.

Motion counteracts the adhesion formation as mechanical stress plays a crucial role in minimizing adhesion formation [23, 30]. Rehabilitation protocols focus on enhancing tendon gliding, which is essential for preventing long-term functional impairment [32, 33]. Early active rehabilitation after surgery, together with the use of shorter splints that spare the wrist, is increasingly recommended to reduce adhesion formation and prevent stiffness in the interphalangeal joints, while passive approaches such as “place-and-hold” protocols are no longer recommended [34, 35]. However, the available evidence remains limited: both the systematic review of Neiduski and Powell and the Cochrane review by Peters et al. emphasized that, despite the frequent claim of superiority of early active mobilization (EAM) protocols, this assumption cannot be substantiated due to heterogeneous, sparse, and poorly reported data, making meaningful comparisons difficult [8, 9]. Consistent with this, the present study found no significant differences in rerupture rates among immobilization, Kleinert, EAM, and Duran-Houser protocols. Likewise, Demirci et al. reported similar functional improvements after active and passive rehabilitation, with no significant differences between groups in overall functional outcomes. Shear wave elastography, used to quantify tendon stiffness, showed no consistent association with clinical parameters and no differences across protocols or time points. However, this study was designed as a pilot trial with a small sample size (*n* = 20) and a short follow-up of 12 weeks, limiting its ability to detect infrequent events such as reruptures [36]. By contrast, the systematic review by Starr et al. demonstrated statistically significant differences: rerupture rates were approximately 4% with early passive mobilization (Kleinert and Duran-Houser) and 5% with early active mobilization. Passive protocols, however, were linked to a higher risk of motion limitations. Overall, rerupture rates have declined over recent decades, which reflects advances in suture techniques and rehabilitation strategies [37]. In summary, rehabilitation after flexor tendon repair remains a subject of ongoing debate. Current evidence highlights the trade-off between rupture risk and postoperative stiffness and underlines the need for high-quality, standardized comparative trials to establish clearer recommendations. In a retrospective study of 291 patients, a surgical delay of more than 14 days showed no significant impact on active range of motion in the DIP and PIP joints. However, including the timing of surgery in the regression model improved its predictive power, suggesting that surgical timing may still play a role in outcomes when considered alongside other factors [18].

In the current study, no significant difference in the occurrence of reruptures could be shown between those acute primary tendon repairs and the delayed primary tendon repair with surgery within two weeks. This supports suturing tendon injuries even if the injury occurred more than a day ago and aligns with findings from previous research, where delays in surgery for FPL repair did not significantly affect functional outcomes [38]. This supports the approach of postponing flexor tendon surgery in selected cases until specialized care is available.

The category of anesthesia did not play a statistically significant role. Operations under regional and general anesthesia had a higher rerupture rate than the WALANT method.

This can be explained by the fact that gapping and bulging, which lead to rupture, can be recognized and immediately corrected intraoperatively by active testing using the WALANT approach [3, 39–41]. The literature, however, is still insufficient to demonstrate a significant difference in the postoperative results between WALANT and traditional anesthesia [3, 39, 40, 42–44]. Except for a markedly elevated infection rate in the WALANT group of Townsend et al. [40], which could not be confirmed by Kadhum et al., the complication and reoperation rates of both groups are similar [40, 42, 44].

Core suture technique (2-strand vs. multi-strand) and peripheral suture type did not significantly affect rerupture rates. This is not in accordance with earlier studies. Biomechanical studies showed the superiority of multiple strand techniques over the double-strand technique in vitro as well as clinical in vivo studies and previous research acknowledges the peripheral circumferential suture as a significant independent factor of tendon repair strength [5, 45–64]. Distinctions could be seen in the rerupture rate after usage of simple running stitches, with a rate of 7.3% (7 in 89 tendons), compared to simple locking stitches, which showed a lower rate of 1.6% (1 in 63 tendons) for the peripheral suture. However, in 177 cases (12 reruptures, 6.8%), the suture technique was not documented, which limited the ability to evaluate these differences with statistical significance (*p* = 0.389). These findings may indicate that while the type of peripheral suture can influence the rerupture rate, its overall impact appears limited in this dataset, particularly due to the high number of undocumented cases. In this context, international recommendations favor six-strand core sutures for FDP repairs in zone II and strong repair techniques in zones III to V. Continuous peripheral sutures should be avoided and replaced by interrupted stitches to close gaps in six-strand repairs. A minimally invasive approach, especially in zones I and II, is advised, and suture knots should be positioned external to the tendon surface [34]. In line with our findings, recent biomechanical data suggest that suture material may not be the primary determinant of repair stability. DYNACORD and FiberWire showed comparable stability under simulated early mobilization. Nevertheless, DYNACORD showed greater shortening within the interweaving zone, indicating improved tissue apposition and reduced gap formation, and may support earlier mobilization with fewer adhesions, although clinical relevance remains uncertain due to cadaveric data [65]. Outcomes of secondary reconstruction further emphasize the importance of optimal primary repair. Unglaub et al. reported adequate function in 75% after two-stage FPL reconstruction (median DASH 11); however, recovery remained inferior to primary repair, with reduced range of motion and notable complication rates. These observations underscore the importance of achieving a stable and well-executed primary tendon repair [66].

Despite advances in flexor tendon surgery, outcomes remain limited in complex injuries, particularly in zone V lesions involving several tendons and in zone I FDP repairs. Therefore, in zone I, many centers favor direct reinsertion using high-strength core sutures [35].

Strengths of this study include the large sample size and the comprehensive analysis of potential risk factors. In particular, injury etiology was investigated for the first time as a possible determinant of outcome, alongside numerous established clinical and surgical variables. This broad approach allows for a more differentiated understanding of factors influencing rerupture risk and functional recovery. This study is limited by its retrospective design, single-center setting, and reliance on electronic health records, which may introduce documentation biases and some lack of specifications. Additionally, long-term functional outcomes were not evaluated, necessitating further prospective studies. Yet, the research question was based on the occurrence of rerupture and potential risk factors and not on the clinical outcome. As mentioned above, the limitation of this study is the small number of reruptures. This particularly affected the multivariate analysis, where additional potential risk factors may not have reached statistical significance due to the limited number of events.

## Conclusion

This study systematically analyzed patient-, injury-, surgery-, and rehabilitation-related risk factors for rerupture following primary flexor tendon repair of the hand and wrist, analyzing 429 complete lesions in 292 patients. While several factors—including sex, age, injury etiology, and pulley reconstruction —were significantly associated with rerupture in univariable analysis, only male sex and pulley reconstruction emerged as independent predictors in the multivariable model. These results underscore the importance of vigilant postoperative management in male patients and careful consideration regarding the necessity and technique of pulley reconstruction or venting in all cases- Moreover, the identification of risk factors for reoperation provides valuable guidance for surgeons and therapists in optimizing treatment strategies for flexor tendon injuries. Given the retrospective single-center design and the limited number of reruptures, further multicenter prospective studies with larger patient cohorts are needed to confirm our findings. Such studies should also incorporate standardized long-term functional outcome measures and more detailed clinical documentation to capture additional explanatory variables. This approach may enable a more comprehensive assessment of patient-, injury-, and surgery-related factors, thereby improving the prediction and prevention of reruptures after flexor tendon repair.

**Table 1:** Rerupture rates in complete versus partial flexor tendon lesions

	Rerupture *n* (%)	No rerupture *n* (%)	*p*-Value
Complete	21 (4.9)	408 (95.1)	< .001^1^
Partial	1 (0.4)	275 (99.6)	
Total	22 (3.1)	683 (96.9)	

^1^Chi-Square Test

**Table 2:** Distribution of documented rerupture causes by time interval after primary repair

Time of rerupture	≤ 6 weeks	7-12 weeks	> 12 weeks
Cause of rerupture: patients (reruptured tendons)			
Incompliance	5 (5)	2 (2)	2 (2)
Incompliance + complex injury + trauma	1 (2)		
Incompliance + infection		1 (1)	
Iatrogenic	1 (2)		
Complex injury of the area	1 (3)		
Trauma		2 (2)	
Minor trauma with underlying rheumatic disease		1 (1)	
Increased scarring			1 (1)
Total: patients (reruptured tendons)	8 (12)	6 (6)	3 (3)

**Table 3:** Rerupture rates after complete flexor tendon repair according to injury-related characteristics

Total	Rerupture *n* (%)	No rerupture *n* (%)	*p*-Value
	21 (4.9)	408 (95.1)	
Injured side			
Left	12 (5.1)	223 (94.9)	.823^1^
Right	9 (4.6)	185 (95.4)	
Etiology of injuries			
Acute: cut or stab	13 (4.3)	292 (95.7)	0.005^3^
Acute: crushed	3 (9.7)	28 (90.3)	
Acute: saw	0 (0.0)	54 (100.0)	
Acute: axe	3 (33.3)	6 (66.7)	
Acute: hyperextension	1 (6.7)	14 (93.3)	
Chronic	1 (20.0)	4 (80.0)	
Other (acute)	0 (0.0)	10 (100.0)	
Closed or open injury			
Open	19 (4.8)	380 (95.2)	.651^2^
Closed	2 (6.7)	28 (93.3)	
Single or multiple injuries			
Single tendon	11 (5.5)	189 (94.5)	.657^2^
Multiple tendons	10 (4.4)	219 (95.6)	
Single ray	12 (4.0)	285 (96.0)	
Multiple rays	9 (6.8)	123 (93.2)	.231^2^
Injured finger			
Thumb	4 (8.2)	45 (91.8)	.775^3^
Index finger	5 (5.1)	94 (94.9)	
Middle finger	4 (5.6)	67 (94.4)	
Ring finger	2 (3.0)	64 (97.0)	
Little finger	5 (5.3)	89 (94.7)	
Wrist	1 (2.0)	49 (98.0)	
Injured zone			
1	4 (6.1)	62 (93.9)	.614^3^
2	11 (5.8)	180 (94.2)	
3	33 (6.0)	47 (94.0)	
4	0 (0.0)	5 (100.0)	
5	3 (2.6)	114 (97.4)	
Injured flexor tendon			
FDP	12 (5.5)	205 (94.5)	.596^3^
FDS	4 (3.3)	116 (96.7)	
FPL	4 (8.3)	44 (91.7)	
FCR	1 (4.0)	24 (96.0)	
FCU	0 (0.0)	19 (100.0)	
Additional injuries			
Yes	7 (3.3)	208 (96.7)	.115^1^
No	14 (6.5)	200 (93.5)	

^1^Chi-Square Test, ^2^Fisher’s Exact Test, ^3^Fisher-Freeman-Halton Exact Test

**Table 4:** Rerupture rates according to timing, anesthesia, and surgical team

	Rerupture *n* (%)	No rerupture *n* (%)	*p*
Time of surgery after injury			
Within one day	18 (5.2)	326 (94.8)	1.000^1^
On day 2-14	3 (4.8)	59 (95.2)	
In week 3-5	0 (0.0)	16 (100.0)	
After week 5	0 (0.0)	7 (100.0)	
Anesthesia			
GA	16 (6.1)	247 (93.9)	.136^3^
RA	3 (5.8)	49 (94.2)	
WA	2 (1.8)	112 (98.2)	
Duration of the surgery			
< 30 min	0 (0.0)	1 (100.0)	.692^1^
30–120 min	13 (4.3)	286 (95.7)	
> 120 min	8 (6.2)	121 (93.8)	
Main surgeon			
Resident	6 (5.9)	95 (94.1)	.599^2^
Consultant	15 (4.6)	313 (95.4)	
Hand surgeon part of the team			
Yes	15 (4.7)	307 (95.3)	.796^2^
No	6 (5.6)	101 (94.4)	
Hand surgeon present is a fully trained specialist			
Yes	11 (4.0)	263 (96.0)	.351^2^
No	10 (6.5)	145 (93.5)	
Total	21 (4.9)	408 (95.1)	

^1^Fisher-Freeman-Halton Exact Test,

^2^Fisher’s Exact Test,

^3^Likelihood Ratio

**Table 5:** Re-rupture rates according to surgical technique and suture-related characteristics

	Rerupture *n* (%)	Without rerupture *n* (%)	*p*-Value
Core suture technique			.735^1^
2-strand technique	8 (5.1)	148 (94.9)	
4-strand technique	7 (6.0)	109 (94.0)	
6-strand technique	3 (7.7)	36 (92.3)	
Other	0 (0.0)	6 (100.0)	
Anchor	0 (0.0)	24 (100.0)	
Transosseous	1 (7.1)	13 (92.9)	
Not documented	2 (2.7)	72 (97.3)	
Peripheral suture			.093^2^
Yes	20 (5.9)	321 (94.1)	
No	1 (1.1)	87 (98.9)	
Pulley reconstruction			.006^2^
Yes	10 (10.9)	82 (98.1)	
No	11 (3.3)	326 (96.7)	
Core suture material			.025^1^
Braided polyblend	16 (7.8)	189 (92.2)	
Braided polyester	3 (5.3)	54 (94.7)	
Other	0 (0.0)	8 (100.0)	
Not documented	2 (1.3)	157 (98.7)	
Core suture diameter			.816^1^
≤ 3-0	0 (0.0)	25 (100.0)	
4-0	5 (5.2)	92 (94.8)	
≥ 5-0	0 (0.0)	3 (100.0)	
Not documented	16 (5.3)	288 (94.7)	
Total	21 (4.9)	408 (95.1)	

^1^Fisher-Freeman-Halton Exact Test

^2^Fisher’s Exact Test

**Table 6:** Re-rupture rate depending on type of peripheral suture

	Rerupture *n*(%)	Without rerupture *n* (%)	*p*-Value
Peripheral suture technique			
SRS	7 (7.3)	89 (92.7)	
SLS	1 (1.6)	63 (98.4)	.420^1^
Other	0 (0.0)	4 (100.0)	
Not documented	12 (6.8)	165 (93.2)	
Peripheral suture material			.378^1^
Nonabsorbable monofilament	14 (5.3)	251 (94.7)	
Other	3 (10.7)	25 (98.3)	
Not documented	3 (6.3)	45 (93.8)	
Peripheral suture diameter			.954^1^
≤ 5-0	4 (4.8)	79 (95.2)	
≥ 6-0	11 (6.3)	164 (93.7)	
Not documented	5 (6.0)	78 (94.0)	
Total	20 (5.9)	321 (94.1)	

^1^Fisher-Freeman-Halton Exact Test

**Table 7:** Binary logistic regression model on the predicted probability for rerupture

	OR (CI 95%)	*p*-Value
Sex		
Female (reference)		
Male	13.235 (1.204-145.449)	0.035
Age (years)		
≤ 18	0.000 (0.000-)	0.997
19-29 (reference)		0.150
30-49	0.127 (0.015 - 1.094)	0.060
50-69	1.021 (0.170-6.152)	0.982
≥ 70	0.729 (0.128-4.150)	0.722
Etiology of injuries		
Acute: cut or stab (reference)		0.375
Acute: crushed	0.989 (0.237-4.123)	0.987.997.067.715.042.999
Acute: saw	0.000 (0.000-)	
Acute: axe	6.772 (0.872-52.576)	
Acute: hyperextension	1.542 (0.151-15.787)	
Chronic	23.261 (1.126-480.480)	
Other (acute)	0.000 (0.000-)	
Pulley reconstruction		
Yes	0.211 (0.073-0.611)	0.004
No (reference)		

*OR* odds ratio,; *CI* confidence interval

## Data Availability

The data supporting the findings of this study are not publicly available due to ethical and data protection restrictions but are available from the corresponding author upon reasonable request.

## Declarations

### Trial registration

Not applicable (retrospective observational study).

### Use of artificial intelligence

Artificial intelligence (AI) was used in the preparation of this manuscript solely to improve clarity and language. Specifically, the GPT-4 language model by OpenAI was used to assist in refining the text, enhancing readability, and ensuring grammatical accuracy. AI was not used for study design, data collection, data analysis, or interpretation.
